# Injuries to the Eye, and Their Medico-Legal Aspect

**Published:** 1878-07

**Authors:** 


*Injuries of the Eye and their Medico-Legal Aspect.* By FERDINAND VON ARLT, M. D., Professor of Ophthalmology in the University of Vienna. Translated with the permission of the author by Charles S. Turnbull, M. D., Surgeon in the Eye and Ear Department, Howard Hospital; chief of the Ear Clinic, Jefferson Medical College Hospital, etc. Philadelphia: Claxton, Remsen & Haffelfinger.

The author states that as a member of the Medico Legal Commission of the University of Vienna, he has noticed a want of a work that presents information upon the subject of injuries of the eye and their treatment, in a more readily accessible form. He has therefore prepared a compend upon the subject in which he does not endeavor particularly to present original material. It cannot but be felt that the distinguished author does good service to the profession, and that his volume will be of value for the purpose for which it is designed.

